# The dose-response relationship between BMI and hypertension based on restricted cubic spline functions in children and adolescents: A cross-sectional study

**DOI:** 10.3389/fpubh.2022.870568

**Published:** 2022-12-19

**Authors:** Yani Wang, Congcong Min, Xiaoyan Song, Heyue Zhang, Chen Yuan, Lizhen Chen, Haiying Zhang

**Affiliations:** ^1^Qingdao Municipal Center for Disease Control and Prevention, Qingdao, China; ^2^Department of Gastroenterology, The Affiliated Hospital of Qingdao University, Qingdao, China; ^3^Health Management Center, Qingdao Central Hospital, Qingdao University, Qingdao, China; ^4^Department of Infectious Disease, Qingdao Municipal Hospital, Qingdao University, Qingdao, China

**Keywords:** hypertension, body mass index, restricted cubic spline, children, adolescents

## Abstract

**Background:**

A high body mass index (BMI) is a major risk factor for hypertension. The purpose of this study was to investigate the association between the BMI and hypertension in children and adolescents.

**Methods:**

We analyzed physical examination data from 29,810 students aged 6–14 years old. A restricted cubic spline (RCS) function was used to investigate the dose-response relationship between the BMI and hypertension.

**Results:**

The prevalence of hypertension was 9.91%, followed by 11.71% in males and 7.9% in females, respectively. Compared to the normal weight group, the odds risk (OR) for hypertension in the overweight group was 1.729, and the OR for hypertension in the obesity group was 3.089. After adjusting for potential confounders, the adjusted ORs were 1.620 [95% confidence interval (CI): 1.457–1.800] in the overweight group and 3.092 (95% CI: 2.824–3.385) in the obesity group. According to the multivariate RCS regression analysis, there was a significant non-linear dose-response association between the BMI and the risk of hypertension (all P-values for non-linear < 0.001).

**Conclusion:**

The dose-response relationship analysis showed that the association strength of hypertension increased non-linearly along with the continuous change of BMI in children and adolescents.

## Introduction

Hypertension is becoming a global public health crisis, which attributes to more than 45% of cardiovascular disease and 51% of stroke deaths (1). What deserves our special attention is that hypertension is not a disease confined to adults, as demonstrated by the fact that more and more children are suffering from hypertension (2). In a cohort study of 38,822 students (19,456 boys and 19,366 girls) aged 7–17 years old in China, the prevalence of hypertension is reported to be 17.00% in boys and 14.13% in girls (3). Recently, a study including 15,143 records of children and adolescents from the China Health and Nutrition Survey 1989–2015, revealed increasing trends in the blood pressure (BP) levels and the prevalence of hypertension (*P* < 0.001) (4). Previous researches have shown that childhood hypertension can lead to early damage of the structure and function of target organs such as the heart and blood vessels, and this damage may subsequently increase the risk of cardiovascular disease in adulthood (5–7). However, the effects of elevated childhood BP are not completely permanent. If high BP has been reversed in childhood before adulthood, the risk of cardiovascular disease will be significantly decreased in the future (8). Moving down the window of hypertension prevention to children and adolescents is a fundamental strategy to curb the rising trend of hypertension and to reduce the burden of cardiovascular disease. Therefore, there is a great significance to identify the risk factors of hypertension and in turn to identify and prevent hypertension in children and adolescents.

At present, some problems such as inconsistent and imperfect standards for adolescent hypertension screening remain exist. In 2010, Mijie et al. (9) developed the first reference standard for gender and age-specific BP of Chinese children aged 3–17 based on national representative data, and wrote it into the “China Guidelines for the Prevention and Treatment of Hypertension 2010” for the first time. It has been widely used in clinical practice (10). This standard tends to consider the convenience of use but not the height factor. In practical application, some tall children will be misdiagnosed.

Accurate indicators for hypertension screening in children and adolescents are emerging. Evidence indicate a potential link between overweight and obesity and the risk of hypertension among children and adolescents (4, 11–14). Ye et al. had reported that the BMI and general obesity were associated with the presence of isolated systolic hypertension, isolated diastolic hypertension, and combined systolic and diastolic hypertension among 15,143 youths (4). Recently, a study of children living in Port Harcourt, Rivers State, Nigeria, showed that overweight/obese children aged 6–16 years old were more susceptible to suffer from hypertension than normal weight and underweight children (*P* < 0.001) (13). Similarly, Taghizadeh et al. have revealed that the BMI is positively correlated with the systolic blood pressure (SBP) and diastolic blood pressure (DBP) (*P* < 0.005 and *P* < 0.007), suggesting that a higher BMI could be a potent predictor of high BP among Iranian children and adolescents (14). However, most studies are limited to multivariate analysis of BMI according to categorical variables (15). Analysis of the dose–response relationship between continuous changes of BMI and the prevalence of hypertension is particularly limited. In this study, the dose–response analysis was conducted to quantify the association between the BMI and hypertension by using the restricted cubic spline (RCS) function. Results would provide significant data for high BP and hypertension intervention of children and adolescents.

## Materials and methods

### Study design

We performed a cross-sectional study using baseline data obtained from Qingdao Central Hospital in Qingdao City, Shandong, China. A total of 30,194 individuals aged 6–14 years old with their physical examination data were recruited from January 2020 to December 2020, which accounted to 4.60% of students aged 6–14 in Qingdao.

After deleting the missing values and the extreme values (BMI > 32), 29,810 subjects of 31 schools (including 23 primary school and 8 junior high school) were finally included. Height, weight, and BP from all participants were measured according to the detailed rules for the investigation and testing of Chinese students' physical health.

The written informed consent for inclusion and data collection before participating in this study were provided from all subjects. The Ethics Committee of Qingdao Central Hospital (Reference Number: KY202109601) approved the study protocol, and this study was conducted in accordance with the principles of the Declaration of Helsinki by the World Medical Association.

### Definitions

Hypertension was defined as a SBP or a DBP greater than or equal to the 95th percentile of those with the same sex, age, and height, according to the updated BP references for Chinese children aged 3–17 years issued by the Chinese Child BP References Collaborative Group (10).

The diagnosis of hypertension was based on 2 separate measurements by using an Omron electronic sphygmomanometer on the right arm after 15 min of seated rest. Students with abnormal BPs on the initial measurement had another repeated measurements to confirm the presence of hypertension. The BMI was calculated as the ratio of the body weight in kilograms and the squared body height in meters (kg/m^2^). The height and weight were measured with light clothing and without shoes by Omron electronic scale. Based on the BMI cut-offs for overweight and obesity in Chinese children and adolescents aged 2–18 years, 29,810 students were sensitively distinguished into three groups: obesity, overweight, and normal (10–17).

### Statistical analysis

Data were analyzed by SAS 9.3 (Cary, New York City, NY, USA). The demographic characteristics of the subjects are presented as numbers and frequency distributions for categorical variables, as appropriate. The SBP or DBP levels of the 95th percentiles at seven ranges of height for each sex were used to select students with hypertension according to the reference (10). Univariate logistic regression analysis was used to determine significant differences between individuals with hypertension and those without hypertension. Unconditional multivariate logistic regression models were performed to investigate the association between the BMI and hypertension in models adjusted for age and sex to evaluating the potential confounding effects. Odds ratios (ORs) and their 95% confidence intervals (CIs) in these different logistic regression models were calculated.

To analyze the dose–response relationship between the BMI and hypertension, we used RCS models fitted for lrm models to adjust age.

The junction between two intervals is called “knot”. Typically, there is a small number of knots (between 3 and 8), which must be specified by the user; let K be the number of knots. We choose the location of 3 knots according to the percentiles (25th, 50th, and 75th) of the distribution of the BMI. The general formula of an unadjusted spline regression is the following (18):


$$\begin{array}{l} Y\left(v\right)=\alpha +\overset{N}{\underset{i=0}{\sum }}\delta iSi\;\left(v\right) \end{array}$$

where *v* is the value of a continuous exposure V, α being the intercept, *Si* representing the spline, δ *i* being the estimate related to the *Si* spline. N is the total number of splines included into the regression, which depends on the number of knots K and the type of spline functions.

The results were presented as OR with their 95% CI. R software (version 4.0.2) was used to carry out the above-mentioned dose–response analysis. A significance level of *P* < 0.05 (two-tailed test) was used.

## Results

### Prevalence of hypertension

The prevalence of hypertension was 9.91% (11.71% for males and 7.9% for females) among recruited 29,810 students. The 25th, 50th, 75th percentiles of BMI were 14.66, 20.44, and 26.22 kg/m^2^, respectively. The highest prevalence of hypertension (31.26%) was found when the BMI was greater than 26.22. The prevalence of hypertension was 15.44, 6.43, and 2.15% when the BMI range was 20.44–26.22, 14.66–20.44, and < 14.66, respectively. Individuals aged 12, 13, or 14 years old had the highest levels of hypertension (24.05, 25.57, and 15.84%, respectively). The descriptive results were listed in Table 1, and significant differences were found for sex, age, and BMI (all *P* < 0.001).

**Table 1:** Characteristics of the study samples (*N* = 29,810).

**Variables**	***N***	**Hypertension (*n*)**	**Rate (%)**	***χ^2^***	***P***
**Gender**					
Male	15,741	1,844	11.71	121.258	< 0.001
Female	14,069	1,111	7.90		
**BMI (kg/m** ^**2**^ **)**					
Normal	16,871	1,090	6.46	690.496	< 0.001
Overweight	5,932	633	10.67		
Obesity	7,007	1,232	17.58		
**Age**					
6	3,755	147	3.91	2092.151	< 0.001
7	3,959	146	3.69		
8	4,165	165	3.96		
9	3,616	196	5.42		
10	3,366	243	7.22		
11	3,095	308	9.95		
12	2,645	636	24.05		
13	2,968	759	25.57		
14	2,241	355	15.84		
Total	29,810	2,955	9.91		

### Univariate and multivariate logistic regression analysis for the association between BMI and hypertension

The association between hypertension and each BMI category was estimated by logistic regression analysis. As shown in Table 2, compared with normal BMI, overweight BMI and obesity BMI were significantly associated with hypertension in both the univariate model and adjusted model. The unadjusted ORs were 1.729 (95% CI: 1.561–1.917) for the overweight BMI and 3.089 (95% CI: 2.832~3.369) for the obesity BMI. After adjusting for age and sex, the adjusted ORs were 1.620 (95%CI: 1.457–1.800) for the overweight BMI and 3.092 (95%CI: 2.824–3,385) for the obesity BMI.

**Table 2:** Logistic regression analyses of the association between BMI and hypertension.

**Model adjustment**	**BMI classification**	**SE**	**Wald**	***P***	**OR (95%CI)**
**Univariate analysis**					
	Normal		647.098	< 0.001	1.00
	Overweight	0.052	109.160	< 0.001	1.729 (1.561–1.917)
	Obesity	0.044	647.000	< 0.001	3.089 (2.832–3.369)
**Adjust model** ^**a**^					
	Normal		598.456	< 0.001	1.00
	Overweight	0.054	80.186	< 0.001	1.620 (1.457–1.800)
	Obesity	0.046	596.714	< 0.001	3.092 (2.824–3.385)

^a^Model a was adjusted for age and gender. OR, odds risk.

### Dose–response relationship between BMI and hypertension

Based on the stratification by sex, the relationship between BMI and hypertension risk was analyzed using the RCS function with 3 knots (The 25th, 50th, 75th percentiles of BMI). There was a significant non-linear dose–response association between the BMI and the risk of hypertension (all *P*-values for non-linear < 0.001). In addition, dose–response relationship analysis showed that the association strength of hypertension increased non-linearly with the continuous change of BMI. These non-linear models were refitted to assess for possible modifications by age.

### Dose–response relationship between BMI and hypertension in males

**Figure 1:**
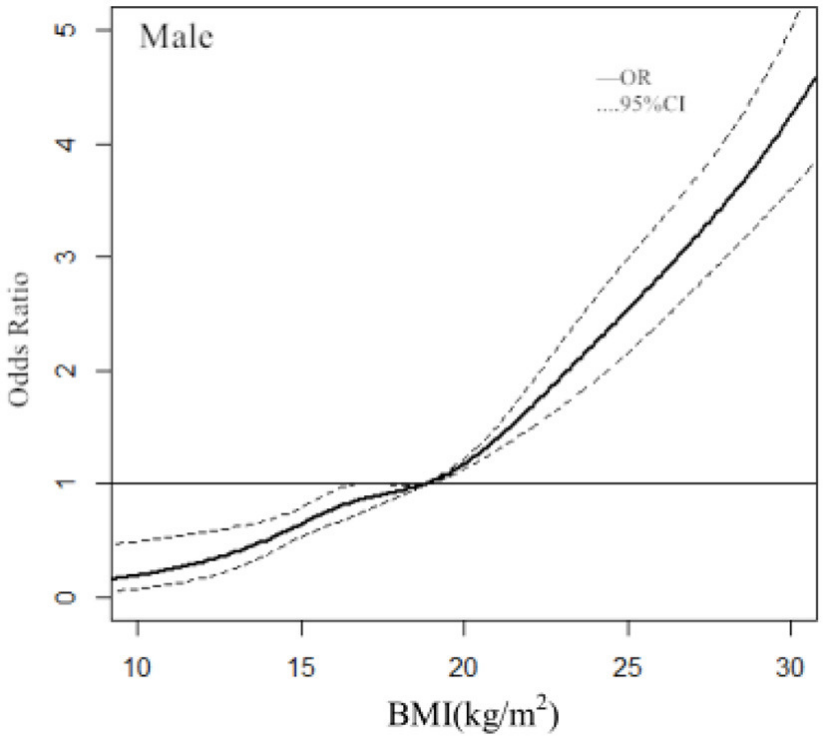
Association between the BMI and hypertension in males, allowing for nonlinear effects, with 95% CIs. The RCS function with 3 knots for BMI, adjusted for age, was performed. Curves show ORs compared with the chosen reference BMI of 18.82 kg/m^2^.

In males, with a BMI of 18.82 kg/m^2^ used as the reference (OR = 1), the point estimates for a BMI of 20.44 or 26.22 kg/m^2^ increased the risk of hypertension [OR (95% CI), 1.27(1.20–1.34) and 2.90 (2.48–3.39), respectively], whereas the point estimate for a BMI of 14.66 kg/m^2^ decreased the risk of hypertension [OR (95% CI), 0.60 (0.48–0.75)] (Figure 1).

### Dose–response relationship between BMI and hypertension in females

**Figure 2:**
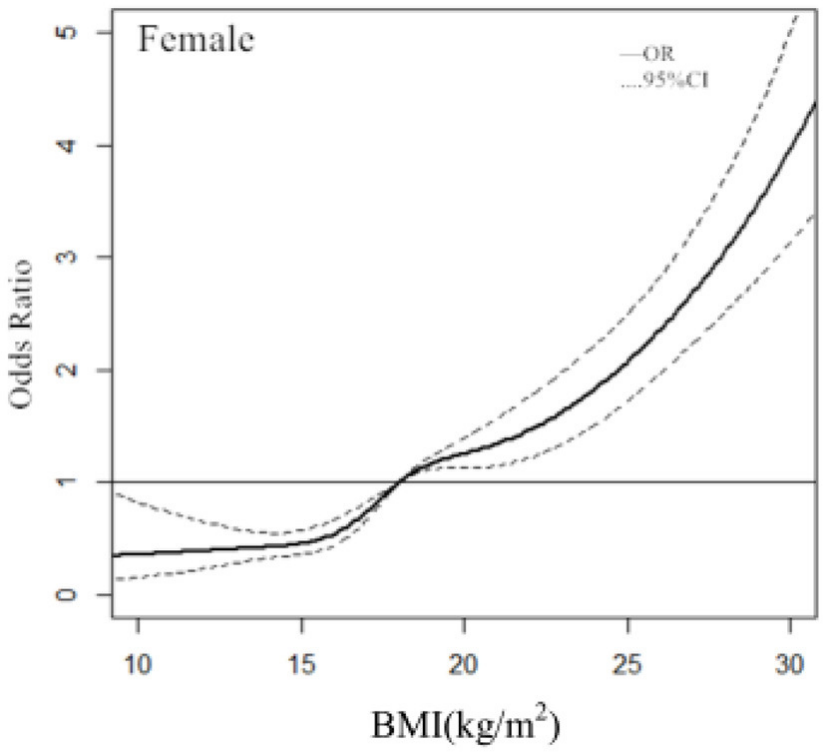
Association between the BMI and hypertension in females, allowing for nonlinear effects, with 95% CIs. The RCS function with 3 knots for BMI, adjusted for age, was performed. Curves show ORs compared with the chosen reference BMI of 18.02 kg/m^2^.

In females, with a BMI of 18.02 kg/m^2^ used as the reference (OR = 1), the ORs and 95% CIs of the RCS function with 3 knots for BMI were 0.44 (0.35–0.55) for 14.66, 1.29 (1.13–1.47) for 20.44 kg/m^2^, and 2.43 (2.02–2.91) for 26.22 kg/m^2^ (Figure 2).

## Discussion

There were several critical findings on the relationship between BMI and hypertension in this study. Firstly, our study suggested that the prevalence of hypertension in the obesity group and the overweight group was significantly higher than that in normal weight group, which was consistent with other studies (4, 11–14). Moreover, our results showed that the risk of hypertension in the overweight group and the obesity group was 1.620 times and 3.092 times that of the normal weight group, respectively (*P* < 0.001). Cheung has reported that although an increasing BMI continues to be strongly predictive of an increase in hypertension prevalence, it appears to have a smaller impact on African American and Asian populations (19). Our results are similar to Cheung's results that the risk of hypertension in the overweight group and the obesity group was 1.26 times and 3.30 times that of the normal weight group in Asian populations (19).

The other finding in this study was the dose-response relationship between BMI and hypertension. Dose-repose curve was applied to provide the continuous ORs of BMI on hypertension. It was pointed out that the ORs of BMI for hypertension increased with BMI. Most studies are limited to multivariate analysis of BMI according to categorical variables (15, 20–22). Our analysis revealed more significant information to reflect the overall trend for the ORs of BMI. Most studies of the dose–response relationship between BMI and hypertension were aimed at adults (23, 24), few studies have been conducted on children and adolescents. RCS analysis showed the clear dose–response relationships between the continuous change of BMI and the risk of hypertension in children and adolescents. Of note, the diagnostic points for BMI classification in children and adolescents were not fixed. Therefore, we used 3 knots at prespecified locations according to the percentiles of the distribution of BMI: the 25th, 50th, and 75th percentiles. We found that the BMI had no effect on the risk of hypertension when BMI ≤ 18.82 kg/m^2^ in males and BMI ≤ 18.02 kg/m^2^ in females, and the risk of hypertension increased significantly when BMI > 18.82 kg/m^2^ in males and BMI > 18.02 kg/m^2^ in females.

An important highlight in this study was that the updated BP references (10) by age and height were useful for identifying hypertensive children. On the one hand, the updated references provide the BP reference values corresponding to 7 height percentiles for each age group, which could eliminate misdiagnosis and missed cases of hypertension due to height difference. On the other hand, the difference between the measurements of qualified electronic sphygmomanometer verified by international standards and mercury sphygmomanometer is < 5 mmHg. The updated references are also applicable to the measurements of electronic sphygmomanometer.

Our study had several limitations, which may also bring some bias to the findings. First, the causal association between BMI and hypertension is not certain because of the cross-sectional design. Second, although the regression model was adjusted for some covariates, uncollected confounding factors such as household income, smoking and drinking status, exercise may also play a role. Third, students included in this survey were all urban students, lack of discussion for rural students, and the findings may be not be suitable for rural students. Fourth, detection bias may have occurred when measuring height, weight, and BP.

Hypertension among children and adolescents is closely related to family genetics, puberty endocrine, obesity, salt/sodium intake, and lack of physical activity. The findings from this study indicate that slim down as much as possible, within the range of normal BMI, and not affecting normal physical development, may be a good suggestion to the primary prevention of hypertension. It is important to strengthen health education on healthy lifestyles among children and adolescents. In addition, regular and accurate BP measurements, scientific monitoring and evaluations, effective prevention and timely treatment of children and adolescents, can also curb the disease burden of adult hypertension.

## Conclusions

The dose–response relationship analysis performed in this study showed that with the continuous change of BMI, the association strength of hypertension increased non-linearly in children and adolescents. Results would provide significant data for high BP and hypertension intervention of children and adolescents.

## Data availability statement

The original contributions presented in the study are included in the article/supplementary material, further inquiries can be directed to the corresponding authors.

## Ethics statement

The studies involving human participants were reviewed and approved by the Ethics Committee of Qingdao Central Hospital (Reference Number: KY202109601) approved the study protocol. Written informed consent to participate in this study was provided by the participants' legal guardian/next of kin. Written informed consent was obtained from the individual(s), and minor(s)' legal guardian/next of kin, for the publication of any potentially identifiable images or data included in this article.

## Author contributions

LC and HaZ: study concept and design. YW, CM, XS, and CY: subjects collection. YW, CM, and HeZ: acquisition and analysis of data. YW: drafting and writing of the manuscript. YW, CM, LC, and HaZ: revision of the manuscript. All authors have read and approved the final manuscript.
